# Burns in prisoners: A 10-year retrospective cohort study from a UK burns unit^✰^

**DOI:** 10.1016/j.jpra.2026.03.042

**Published:** 2026-04-02

**Authors:** Suraya Mohamed Yusuf, Olivia Hartrick, Bryant Chong, Ella Anthony, Alexander James Baldwin, Alexandra Murray

**Affiliations:** a Department of Burns and Plastic Surgery, Stoke Mandeville Hospital, Buckinghamshire Healthcare NHS Trust, Mandeville Rd, Aylesbury, Buckinghamshire, United Kingdom; b Nuffield Department of Orthopaedics, Rheumatology and Musculoskeletal Science (NDORMS), Botnar Institute of Musculoskeletal Sciences, University of Oxford, Oxford, Oxfordshire, United Kingdom; c Department of Burns and Plastic Surgery, Manchester University NHS Foundation Trust, Oxford Rd, Manchester, United Kingdom; d Faculty of Biology, Medicine and Health, Stopford Building, University of Manchester, Oxford Rd, Manchester, United Kingdom

**Keywords:** Burns, Prisoners, Prisons, First aid, Community health services

## Abstract

**Background:**

Burns among prisoners are complex to manage. Barriers to timely first aid and specialist care may contribute to worse outcomes, yet data on this population remain limited. This study characterized the epidemiology, injury patterns, first aid provision and follow-up engagement of prisoners with burns, to inform improvements to service provision.

**Methods:**

We conducted a 10-year retrospective cohort study of all prisoners treated for burn injuries at a UK regional burns unit between March 2015 and April 2025. Data were extracted from medical records, including demographics, injury characteristics, mechanism and intent, first aid, surgical intervention, complications, and follow-up.

**Results:**

63 cases met inclusion criteria. Median age was 30 years (IQR 24–39), and median TBSA burned was 1.0% (IQR 0.7–2.9). Most burns were scalds (83%), resulting from assault (54%) or self-harm (11%). Assault-related injuries were exclusively scalds and differed significantly in mechanism from self-harm (*p* < 0.001) and accidental injuries (*p* = 0.03). Psychiatric co-morbidity was recorded in 42% of cases, with significant differences between self-inflicted injuries vs. assault-related (*p* = 0.002) or accidental injury (*p* = 0.005). Adequate first aid was documented in only 32% of cases; 30% of referrals were delayed by more than 2 days from injury. Only 63% of prisoners attended scheduled follow-up; missed appointments were often due to institutional constraints.

**Conclusion:**

Burns in prisoners frequently arise from deliberate harm, with high prevalence of psychiatric co-morbidities. They are often inadequately managed in the early phase and poorly followed up. Focus on scald prevention, improved first-aid training, integrated mental health care, and expansion of outreach services may improve outcomes in this high-risk, underserved population.

## Introduction

Prisoners with burn injuries face systemic healthcare inequalities that may contribute to delayed care and poorer outcomes, however this remains under-researched. The global James Lind Alliance Priority Setting Partnership (JLA PSP) for burns, published in 2025, highlights several key research priorities highly pertinent to this group.^1^ These include delivery of equitable care in resource-limited settings, psychological support for affected individuals as well as the education and training of those involved in burns care in this group.

While the prevalence of burns in the general population is well-characterized, data on the incidence and characteristics of burns amongst prisoners remain sparse, despite the distinct challenges this group poses in both the acute and rehabilitative phases of care.^2^, ^3^^–^^4^ Prisoners are exposed to a unique combination of environmental stressors, interpersonal violence, and self-injurious behaviors. Burn injuries sustained in prison occur within a highly constrained institutional environment. Factors such as restricted access to immediate first aid, delays in injury detection, limited staff training in burn management, security-driven delays in transfer, and a high prevalence of psychiatric illness and substance misuse may all influence injury severity, infection risk, and engagement with follow-up care. In our region, prisons referring to the burns service are predominantly Category B and Category C prisons, which house individuals who do not require the highest level of security but for whom escape must be made difficult (Category B), or who cannot yet be trusted in open conditions (Category C). These security classifications necessitate controlled prisoner movement and escort availability, which may further impede timely access to specialist healthcare. The impact of these systemic delays on burn depth progression, infection risk, and long-term outcomes is not well understood.

This study presents a 10-year retrospective cohort study of prisoners treated for burn injuries in our NHS specialized regional burns unit. It aims to characterize the demographic and clinical features of this population, explore injury mechanisms and intent, and evaluate the adequacy and source of initial first aid. We also investigate patterns in referral, treatment, and follow-up, with the objective of identifying modifiable gaps in care that could inform service improvement at the interface between prisons and specialist burn services. To our knowledge, this represents one of the largest studies of its kind, expanding substantially on the existing literature and offering a contemporary overview of burn care within this high-risk, underserved population.

## Methods

A retrospective cohort study was conducted at a regional burns unit in the United Kingdom in accordance with the STROBE guidelines.^5^ The study included all prisoners who sustained burn injuries and were treated at the unit over a 10-year period, from March 2015 to April 2025.

Eligible patients were identified through interrogation of our local, prospectively gathered database of burns referrals, which is used to inform the International Burn Injury Database (iBID). Inclusion criteria were patients who (1) suffered a burn injury assessed and treated by our regional unit and (2) were confirmed to be in prison at the time of injury were included. Cases were cross-referenced with electronic patient records to verify inclusion criteria and ensure data completeness. Records lacking sufficient information to confirm eligibility were excluded from analysis. There were no other exclusion criteria.

Clinical data were extracted through retrospective review of the full medical records, including electronic notes, admission documents, operative records, and follow-up clinic correspondence. Data were extracted and cross-checked by a second reviewer. Variables collected included patient demographics, burn characteristics, treatment, complications and follow-up.

### Data analysis

Descriptive statistics were used to summarize demographic, injury, and treatment characteristics. The Shapiro-Wilk test, used to assess data distribution, demonstrated that all continuous variables were non-normally distributed. As such, continuous variables, including age and total body surface area (TBSA), were expressed as median with interquartile range (IQR) and comparisons across injury subgroups (accidental, assault, self-inflicted) were performed using the Kruskal-Wallis H test. Categorical variables, including burn depth, adequacy of first aid, and the presence of psychiatric comorbidity or intoxication were analyzed using Fisher’s Exact Test due to small sample sizes. For any categorical variable with a significant overall Fisher’s test, post-hoc pairwise Fisher’s exact tests were then performed to identify which specific subgroup pairs differed. All statistical tests were two-sided, with statistical significance (alpha) set at *p* < 0.05. Analyses were performed using R (version 4.4.0, R Foundation for Statistical Computing, Vienna, Austria).

#### Ethical considerations

This study was registered as a service evaluation with the local clinical governance department. As the project involved analysis of routinely collected, anonymized data with no direct patient contact or intervention, formal ethical approval was not required in accordance with Health Research Authority guidance.^6^

## Results

### Demographics and etiology

**Figure 1:**
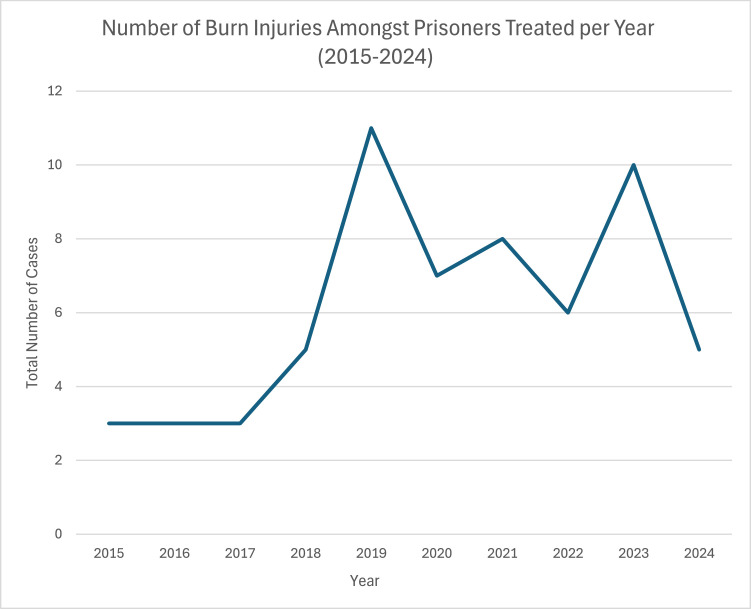
Number of burn cases referred to our regional burns unit over time. Data from 2025 were excluded from this figure due to incomplete year data.

Seventy-four burns were recorded in the prison setting in the 10-year study period. Six patients were excluded as they were staff or otherwise not prisoners, five patients were excluded due to incomplete medical records. Sixty-three cases met inclusion criteria. The temporal spread of these cases is shown in Figure 1 (2025 excluded). The median age was 30 years (Range 17–56 years; IQR 24–39 years). There was one female patient.

### Injury details

The median %TBSA was 1.0 (IQR 0.7, 3.0); mean %TBSA was 2.1 (SD 2.3). Most burns were superficial partial thickness, with 5 patients sustaining deep dermal and 3 patients with full thickness burns. Most burns were scalds (83%), often in the context of violence or self-harm; accidental injuries only accounted for 29% of cases. Whether a burn was as a result of self-harm, assault or accidental did not affect the TBSA (*p* = 0.7) or depth of burn (*p* = 0.11) (Table 2).

### Presentation and first aid

First aid was attempted in 35/63 cases, however adequate first aid was only recorded in 20/63 (32%) of cases. Documentation of who provided first aid to prisoners was limited, however it can be seen that attempts at first aid were suboptimal regardless of the provider: prison officers (4/6 deemed adequate); prison medical staff (2/3 adequate); emergency responders (1/2 adequate); self-administered (0/2 adequate). Inadequate first aid included use of wet towels or running water for limited duration. Nineteen out of 63 patients were referred ≥2 days after their injury (range 2–16 days). Of these, six patients already had signs of infection on first review, with one patient treated for sepsis.

### Outcomes and follow up

Of the 63 cases assessed 12 required surgical intervention, ranging from debridement and dressing, to split thickness skin grafts and local flaps in the most severe cases. Fifteen patients required admission, the median length of stay was 4 days (range 1–23 days).

Nineteen patients experienced delayed referral (>48 h), of whom six (31.6%) developed wound infection, compared with two of 44 patients (4.5%) referred within 48 h of injury. Delayed referral was associated with a significantly higher risk of infection (Fisher’s exact test, *p* = 0.007). Bacteria isolated from infected burns included *Staphylococcus aureus* (*n* = 5) including one case of methicillin-resistant *Staphylococcus aureus, Enterobacter kobei with AmpC activity* (*n* = 1), *Citrobacter koseri* (*n* = 2), *Enterobacter hormaechei* (*n* = 1), *Enterococcus faecalis* (*n* = 1).

46 patients were referred for outpatient follow up in the burn clinic, of which 37% of patients did not attend their scheduled appointments. On several occasions these appointments were canceled by the prison due to limited capacity to accompany patients. Two prisoners were released before follow-up was complete and were lost to follow up. Three patients were referred to scar clinic for long term follow up but never attended. The mean length of follow up for patients 27 patients who had completed follow up at the time of data collection was 13 days (SD 16). The characteristics and outcomes of burns injuries in our patients are summarized in Table 1.

**Table 1:** Characteristics and outcomes of prisoner burn injuries.

Characteristic	Number of patients
	*N* = 63
Age, years, median (IQR)	30 (24, 39)
Sex, n (%)	
Male	62 (98%)
Female	1 (1.6%)
TBSA, %, median (IQR)	1.00 (0.60, 2.90)
Depth of burn, n (%)	
SPT	55 (87.3%)
DD	5 (7.9%)
FT	3 (4.8%)
Type of burn, n (%)	
Chemical	1 (1.6%)
Contact	6 (9.5%)
Flame	4 (6.3%)
Scald	52 (83%)
Mechanism of burn, n (%)	
Accidental	18 (29%)
Assault	34 (54%)
Self-inflicted	7 (11%)
Unknown	4 (6.3%)
Adequate first aid provided, n (%)	
Yes	20 (32%)
No	34 (54%)
Unclear	9 (14%)
Surgery, n (%)	
Debride and dress	6 (9.5%)
Debride and SSG	4 (6.3%)
Debride and terminalization of digit	1 (1.6%)
Debride + dermal skin substitute + SSG + local flap	1 (1.6%)
Not required	51 (81%)
Outpatient follow-up requested, n (%)	46 (73%)
Outpatient follow-up attended (of those requested), n (%)	29 (63%)

TBSA, total body surface area; SPT, superficial partial thickness; DD, deep dermal; FT, full thickness; SSG, split thickness skin graft.

### Subgroup analysis by mechanism of injury

Over half of the included patients sustained burns as a result of assault by other inmates (56%), whilst 10% were self-inflicted (deliberate self-harm through scalding with boiling water, fires in cells, or contact burns), and 28% were due to accidental injury. Four cases had an unclear history, 75% of which were associated with substance use, where the patient could not recall, or refused to disclose, the mechanism of injury. Patient, injury and outcome characteristics were compared according to the mechanism of injury, as summarized in Table 2. For variables with a significant overall Fisher’s test, the results of post-hoc pairwise comparison are shown in Table 3.

**Table 2:** Comparison of selected characteristics by burn category.

Variable	Burn category			*p*-value^a^
	accidental *N* = 18	Self-inflicted *N* = 7	Assault *N* = 34	
Age, years, median (IQR)	34 (30, 43)	28 (25, 47)	25 (21, 36)	0.023
Sex (%)				>0.99
Male	18 (100%)	7 (100%)	33 (97%)	
Female	0 (0%)	0 (0%)	1 (2.9%)	
Type of burn (%)				<0.001
Scald	13 (72%)	3 (43%)	34 (100%)	
Contact	3 (17%)	2 (29%)	0 (0%)	
Chemical	1 (5.6%)	0 (0%)	0 (0%)	
Flame	1 (5.6%)	2 (29%)	0 (0%)	
TBSA, %, median (IQR)	1.00 (0.80, 2.00)	1.20 (0.50, 6.00)	1.40 (0.80, 3.00)	0.70
Depth of burn (%)				0.11
SF	13 (72%)	7 (100%)	32 (94%)	
DD	4 (22%)	0 (0%)	1 (2.9%)	
FT	1 (5.6%)	0 (0%)	1 (2.9%)	
Adequate first aid, *n* (%) of Yes				0.44
Yes	5 (28%)	1 (14%)	13 (38%)	
Substance abuse, *n* (%) of Yes				0.018
Yes	2 (11%)	2 (29%)	0 (0%)	
Psychiatric comorbidities, *n* (%) of Yes				0.004
Yes	6 (33%)	7 (100%)	12 (35%)	
Surgery required (%)				0.014
Yes	3 (17%)	4 (57%)	3 (9%)	
Outpatient follow up requested (%)				0.48
Yes	15 (83%)	5 (71%)	23 (68%)	
Outpatient follow up attended (of those requested) (%)				0.013
Yes	6 (40%)	2 (40%)	19 (83%)	

a Kruskal-Wallis rank sum test; Fisher’s exact test.

**Table 3:** Post-hoc pairwise Fisher’s exact tests.

Comparison by variable	*p*-value
Type of burn	
Accidental: Self-inflicted	0.291
Accidental: Assault	0.003
Self-Inflicted: Assault	<0.001
Substance abuse	
Accidental: Self-inflicted	0.677
Accidental: Assault	0.037
Self-Inflicted: Assault	0.026
Psychiatric comorbidity	
Accidental: Self-inflicted	0.005
Accidental: Assault	1.000
Self-Inflicted: Assault	0.002
Surgery	
Accidental: Self-inflicted	0.133
Accidental: Assault	0.405
Self-Inflicted: Assault	0.030
Outpatient follow up attended (of those requested)	
Accidental: Self-inflicted	1.000
Accidental: Assault	0.040
Self-Inflicted: Assault	0.165

The type of burn (scald, contact, flame or chemical) differed between groups (*p* < 0.001). Assault-related injuries were exclusively due to scalds, in contrast to the self-inflicted group (*p* < 0.001) and accidental group (*p* = 0.003), which demonstrated a broader distribution of etiologies—including contact and flame burns in both, and a chemical burn in the accidental group. No difference in distribution of burn etiology was observed between accidental and self-inflicted burns (*p* = 0.29).

Substance abuse also varied across groups (*p* = 0.009). No cases of assault were associated with documented intoxication, which contrasted with the 29% (*p* = 0.026) of patients with self-inflicted burns and 11% (*p* = 0.037) of patients with accidental injuries who were under the influence of illicit substances, including spice and heroin, at the time of injury.

Likewise, the overall presence of psychiatric comorbidity showed marked difference between groups (*p* = 0.004). All patients with self-inflicted injuries had documented psychiatric comorbidities, compared with 33% (*p* = 0.005) of those with accidental burns and 35% (*p* = 0.002) of those with assault-related burns. No difference was observed between the accidental and assault groups (*p* = 1.000).

Patient with self-inflicted injuries were more likely to require surgical intervention than victims of assault (*p* = 0.03). There was no difference in the proportion of patients who required outpatient follow up across subgroups, however victims of assault were more likely to attend follow up than patients who sustained accidental injuries (*p* = 0.04).

## Discussion

Existing literature on this patient group is limited to small retrospective series. A 1996 report by Lee et al. was the first to highlight the burden of major burns in inmates, raising concerns around staff safety and continuity of care.^2^ Jawad et al. described 18 cases in a single center, emphasizing the predominance of assault-related scald injuries and challenges in arranging adequate outpatient follow-up.^3^ Stone et al. reported similar findings from a 22 patient series of scalds in prisoners.^7^ Rafie et al. extended this understanding with a larger 68-patient cohort, reporting high rates of non-accidental injuries, variable first aid practices, and suboptimal engagement with post-discharge care.^4^

Despite these contributions, our understanding of the epidemiology, prehospital response, and management of burns in prisoners remains incomplete. In particular, the quality and timing of first aid—an intervention known to significantly affect burn depth and healing—has received limited attention.^8^ Additionally, there are few data on who administers this first aid within prison settings, or how variations in provider training and environmental constraints might influence outcomes.

Even relatively small burn injuries assume disproportionate clinical significance within prisons. Inadequate hygiene and difficulty achieving frequent, sterile dressing changes—driven by limited wound-care training and restricted access to healthcare professionals—create conditions in which superficial burns may rapidly become contaminated. Delayed referral to specialist services is particularly consequential; in our cohort, referral beyond 48 h was associated with a markedly higher rate of wound infection. These vulnerabilities are further compounded by poor adherence to outpatient follow-up following discharge, limiting opportunities for early intervention and prolonging healing.

The provision of adequate first aid was notably low across all groups, with fewer than one in three patients receiving optimal early management. The low rates of first aid provision are of particular concern given that early intervention can substantially influence long-term outcomes, reducing both the need for surgical intervention and the extent of permanent scarring.^8^ The prison setting may present particular challenges to timely first aid delivery, whether due to reluctance of victims to seek help, delays in detection, access to appropriate facilities, or limited staff training in burn management protocols. Whilst first aid was attempted in 35 cases, it was only adequate in 20 of these, emphasizing the need for improved education for care providers in immediate burn management. Interestingly, 2 of our patients attempted their own first aid with cooling water, however this was for a limited duration (<5 min) in both cases. This highlights that education should equally be aimed at prisoners themselves, as self- or peer-administered first aid may be especially impactful in cases of delayed presentation following assault.

This study confirms that burn injuries among prisoners predominantly result from deliberate harm, with assaults accounting for over half of all cases and scalds being the most common injury mechanism across all categories. The frequent use of hot liquids, often involving boiling water or substances such as “prison napalm” (boiling sugar solution), remains a recurrent and troubling pattern of assault within this environment, mirroring previous observations in UK literature.^4^ A similar pattern of a significantly higher prevalence of scald injuries in prisons has been demonstrated in the United States, however literature from the wider international population is lacking.^9^ The addition of sugar to boiling water to create “prison napalm” has been shown to increase the depth of burns as tissue spends five times longer exposed to temperatures capable of causing protein denaturation.^10^ The confined setting of the prison creates a context where readily accessible implements such as kettles, sanctioned for personal use, may be weaponized with devastating consequences.

The use of illicit substances such as heroin, cannabis and cocaine has been a documented issue in prisons, with rates of drug use in prison being reported at 40–75%.^11^^,^^12^ Intoxication of prisoners may promote engagement in risky behaviors, increasing the risk of burns, as well as delaying presentation and delivery of first aid, through fear of repercussions or impaired consciousness. Similarly, several reports have highlighted the extremely high prevalence of psychiatric disorders and self-harm in prisoners, calling for reform in the delivery of mental healthcare to these at-risk individuals.^12^^,^^13^ The high prevalence of psychiatric comorbidities in our study was striking, particularly among those sustaining self-inflicted injuries, where all individuals carried a formal diagnosis. This reflects the intersection of mental health vulnerability and risk of burn injury within prison populations. Even among those with accidental or assault-related injuries, the burden of psychiatric morbidity was considerable, highlighting the wider challenges of prison healthcare and the importance of integrated mental health support alongside burn care.

Following the “Patient or Prisoner” report published by the Her Majesty’s Inspectorate of Prisons in 1996, the National Health Service (NHS) assumed responsibility for delivery of health care to prisons.^14^ Despite this, evidence continues to show that whilst prisoners have higher burdens of physical and mental health concerns, they have poor access to health services. A report drawing on over 110,000 patient hospital records for prisoners in England demonstrated that prisoners had 24% fewer inpatient admissions and outpatient attendances than the equivalent age and sex demographic in the wider population.^15^ Data from a later Nuffield trust report published in 2021 suggests the pandemic has worsened access to health care further, with falling rates of hospital admission from March 2020.^16^ This may have been reflected in the dip in burns referrals seen from 2020–2023 in our unit, where injuries may have been treated locally without referral to our service. Importantly the report also found that prisoners missed 42% of outpatient appointments, compared to 23% of the general population.^16^ This is in keeping with the 37% of prisoners who did not attend follow up appointments with our burn clinic. A recent report by the Health Services Safety Investigation Body similarly found “did not attend” (DNA) rates of 43% and 48% for males and females respectively.^17^ They found that prisoners are likely to miss appointments due to the prison regime and logistics beyond their control, for example lack of transport or staff available to escort patients. Interestingly, they also reported a decline in the use of telemedicine appointments since the pandemic.^17^

A systematic review assessing the impacts and outcomes of telehealth delivered in prisons, drawing on data from the UK, US, Australia, China, South Korea and Spain showed that implementation of telehealth was seen to improve access to healthcare.^18^ Seven of 10 included papers reported prisoners were generally satisfied with telehealth consultations. For prisoners in all included studies there was a preference for, or no significant difference between in person, and telehealth appointments.

### Implications for practice

The burns outreach service has the potential to play a critical role in facilitating care for these patients. Our outreach nurses have delivered training to local prison officers and are in the process of establishing an integrated burns clinic within local prisons. This has the potential to provide both continuity of care and logistical flexibility, allowing follow-up to be delivered within the prison estate and reducing the operational burden associated with hospital attendance. In the context of the recognized barriers to outpatient follow-up among prisoners, including difficulties in securing escort staff and transport logistics, the expansion of outreach and telemedicine services may represent a pragmatic and scalable approach to ensuring equitable care for this vulnerable population.

### Limitations

This study has several limitations. Its retrospective nature limits the ability to infer causality. Additionally, the analysis is dependent on the quality and completeness of the original clinical documentation. In particular, details regarding the adequacy of first aid, the presence of complications, and the reasons for missed follow-up were sometimes poorly recorded. Patients were often discharged back to the care of the prison GP before their wounds were fully healed, therefore there is a lack of long term follow up, or any patient reported outcome measures. Some variables—such as psychiatric diagnoses and substance misuse—rely on clinical documentation and may underestimate true prevalence. Subgroup analyses were underpowered due to small sample sizes increasing the risk of type I error, these should be interpreted as hypothesis-generating rather than confirmatory. The single-center design may limit generalizability to other prison populations or burns services.

Future research should incorporate mixed methods approaches, including surveys of prison staff and inmates to identify modifiable risk factors for burn injuries and identify opportunities to improve first aid education and follow up compliance. The implementation of an outreach nurse-led clinic within prisons offers a unique platform to monitor long-term physical outcomes, such as scarring, contractures and infection, as well as the psychological sequelae of burn trauma in this high-risk population.

## Conclusion

This represents one of the largest studies of burns injuries amongst prisoners. Most injuries were superficial with a low TBSA but inadequately managed in the early phase. Clinicians should remain vigilant to the significant overlap between burn injuries, mental health diagnoses, and substance misuse. This highlights the opportunity for integrated, multidisciplinary care models where opportunistic interventions for detoxification and psychological support can be delivered. The prison population represents an extreme example of a resource-limited environment where alternative approaches to care delivery, including training of non-medical personnel, telemedicine and nurse-led outreach clinics, may offer meaningful improvement in both acute care and long-term outcomes.

## Author contribution statement

SY was the primary author, involved in conceptualization, data collection and analysis, and prepared the original draft of the manuscript. BC and EA were involved in data collection. EA provided clinical insight into our burns services. OH, AB and AM were involved with conceptualization and supervised the writing of this manuscript. All authors edited and approved the final manuscript.

## Funding

This research did not receive any grants from funding agencies in the public, commercial, or not-for-profit sectors.

## Declaration of competing interest

The authors have no conflicting interests to disclose.
